# Depression As an Independent Predictor of Nonadherence to Cardiac Rehabilitation in a Latin American Country

**DOI:** 10.1016/j.cjco.2026.02.023

**Published:** 2026-03-19

**Authors:** Hernán G. Rincón-Hoyos, Juan Esteban Aponte-Arroyo, María Cardozo Rengifo, Roger Figueroa Paz, Gabriela Caicedo Samboní, Valentina Calderón Sánchez, Salomé Cardona Giraldo, Ángela Murillo, Orlando Quintero Florez, Juan Carlos Rivas Nieto

**Affiliations:** a Fundación Valle del Lili, Departament of Psychiatry, Fundación Valle del Lili, Cali, Colombia; b Faculty of Health Sciences, Universidad Icesi, Cali, Colombia; c Clinical Research Center, Fundación Valle del Lili, Cali, Colombia; d Physical Medicine and Rehabilitation, Fundación Valle del Lili, Cali, Colombia; e Psychiatric Hospital Universitario Del Valle, Cali, Colombia

**Keywords:** cardiac rehabilitation, depression, anxiety, adherence

## Abstract

**Background:**

Cardiovascular diseases are the leading cause of mortality worldwide, and the primary cause of death from noncommunicable diseases in Colombia. Cardiac rehabilitation programs (CRPs) are effective strategies to reduce cardiovascular mortality, improve functional capacity, and decrease healthcare costs. However, their success depends on patient adherence, which remains suboptimal, particularly in low- and middle-income countries where CRPs are underutilized and understudied. The prognostic impact of anxiety and depression on adherence remains largely unexplored in such countries, where socioeconomic, cultural, and healthcare access barriers may amplify their impact.

**Methods:**

We conducted a retrospective cohort study including patients aged ≥ 18 years who entered a CRP at a Colombian teaching hospital, between 2004 and 2014. Baseline anxiety and depression were measured using the Hospital Anxiety and Depression Scale. Adherence was defined as attending ≥ 30 of 36 sessions. Nested logistic regression models examined the prognostic association between anxiety, depression, and nonadherence, adjusting for demographic and clinical variables.

**Results:**

Among 3659 patients (median age 62 years; 66.2% male), overall nonadherence reached 51.1%. Anxiety was present in 27.9% of patients, and depression in 16.5%. Depression independently predicted nonadherence (odds ratio 1.14; 95% confidence interval: 1.00-1.31), after adjustment for confounders. Anxiety lost its statistical significance in adjusted models (odds ratio 1.06; 95% confidence interval: 0.95-1.19).

**Conclusions:**

Depression at program entry is a modifiable independent predictor of nonadherence in a Latin American CRP. Routine psychological screening and early management of depressive symptoms could enhance adherence and optimize CRPs' effectiveness in resource-limited settings.

## Central Illustration

**Figure:**
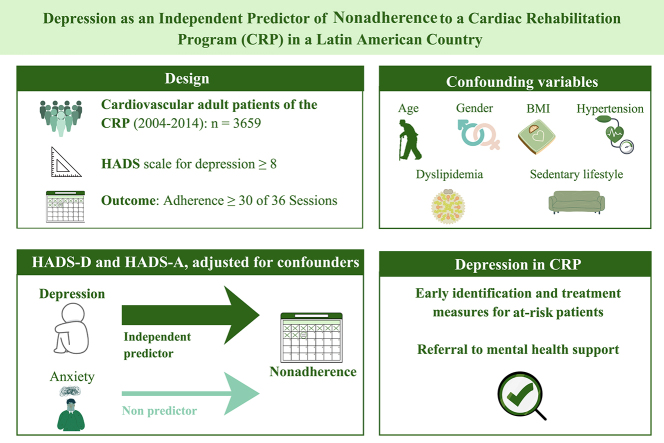

## Résumé

**Contexte:**

Les maladies cardiovasculaires constituent la première cause de mortalité dans le monde et la principale cause de décès par maladie non transmissible en Colombie. Les programmes de réadaptation cardiaque (PRC) sont des stratégies efficaces pour réduire la mortalité cardiovasculaire, améliorer la capacité fonctionnelle et réduire les coûts de santé. Cependant, leur succès dépend de l’observance des patients, qui reste insuffisante, en particulier dans les pays à revenu faible ou intermédiaire (PRFI) où les PRC sont sous-utilisés et peu étudiés. L'impact pronostique de l'anxiété et de la dépression sur l'observance reste largement inexploré dans les PRFI, où les obstacles socio-économiques, culturels et d'accès aux soins de santé peuvent amplifier leur impact.

**Méthodologie:**

Nous avons mené une étude de cohorte rétrospective incluant des patients âgés ≥ 18 ans ayant intégré un PRC dans un hôpital universitaire colombien entre 2004 et 2014. L'anxiété et la dépression initiales ont été mesurées à l'aide d'une échelle quantifiant l'anxiété et la dépression en milieu hospitalier (HADS). L'observance a été définie comme la participation à ≥ 30 des 36 séances. Des modèles de régression logistique imbriqués ont examiné l'association pronostique entre l'anxiété, la dépression et la non-observance, en ajustant sur les variables démographiques et cliniques.

**Résultats:**

Parmi 3 659 patients (âge médian 62 ans; 66,2 % d'hommes). Dans l'ensemble, la non-observance a atteint 51,1 %. L'anxiété était présente chez 27,9 % des patients et la dépression chez 16,5 %. La dépression prédisait de manière indépendante la non-observance (rapport de cotes [RC] 1,14; intervalle de confiance [IC] à 95 % : 1,00–1,31), après ajustement pour les facteurs de confusion. L'anxiété a perdu sa signification statistique dans les modèles ajustés (RC 1,06; IC à 95 % : 0,95–1,19).

**Conclusions:**

La dépression à l'entrée dans le programme est un facteur prédictif indépendant et modifiable de la non-observance dans un PRC en Amérique latine. Un dépistage psychologique systématique et une prise en charge précoce des symptômes dépressifs pourraient améliorer l'observance et optimiser l'efficacité des PRC dans les contextes aux ressources limitées.

Cardiovascular disease (CVD) is the leading cause of morbidity and mortality due to noncommunicable diseases in adults worldwide.^1^ Survivors of cardiovascular events face an elevated risk of recurrence, physical and psychological disability, decline in quality of life, and reduced work productivity.^2^

Cardiac rehabilitation programs (CRPs) are structured, multicomponent interventions that usually include healthy lifestyle education, risk-factor control, psychosocial counselling, and supervised exercise training.^3^ CRPs are recognized and recommended as a secondary prevention strategy for the management of various heart diseases^4^^,^^5^ with evidence supporting their effectiveness in improving cardiac function, reducing relapses, and facilitating a successful return to work.^6^

Although CRPs have been proven effective worldwide, adherence rates vary widely, ranging from 35% to 89% across different contexts and countries.^5^ However, some studies have reported even lower rates. For example, Peters and Keeley^7^ found that only 1 in 3 participants remained engaged, and Kim et al.^8^ reported that 1 in 6 patients adhered to CRPs. In Latin America, adherence is also suboptimal, as fewer than 25% of eligible patients start the program, and approximately half of them discontinue it prematurely.^4^^,^^9^, ^10^, ^11^

Adherence to CRPs is influenced by both psychosocial and clinical factors. Psychosocial factors include gender, ethnicity, cost and accessibility of services, educational level, and employment status.^7^^,^^12^^,^^13^ Clinical factors include comorbidities, fragility, cognitive impairment, and presence of affective disorders, such as depression and anxiety.^9^^,^^13^

When anxiety or depression is present in patients with CVD, these conditions are associated with worse outcomes, including lower adherence to pharmacologic treatment and reduced adoption of healthy lifestyle behaviours.^14^^,^^15^ Most of this evidence comes from high-income countries, where health systems and access barriers differ from those in low- and middle-income countries (LMICs), including much of Latin America.^5^^,^^15^ In this sense, in some middle-income Latin American countries, depression has been reported to be prevalent and linked to increased mortality after a cardiovascular event.^16^

In Colombia, local factors such as limitations in health coverage, geographic and psychosocial barriers, and differences in management of mental health disorders, may influence the relationship between affective disorders and adherence to CRPs.^11^^,^^14^^,^^17^ Therefore, a need remains to generate evidence to support the implementation of intervention strategies aimed at optimizing adherence and improving CRPs outcomes.^9^ This study aimed to determine whether a prognostic association exists between anxiety or depression and nonadherence to cardiac rehabilitation (CR) in patients treated in a CRP at a teaching hospital in southwestern Colombia (CRP-COL) between 2004 and 2014.

## Methods

### Participants

**Figure 1:**
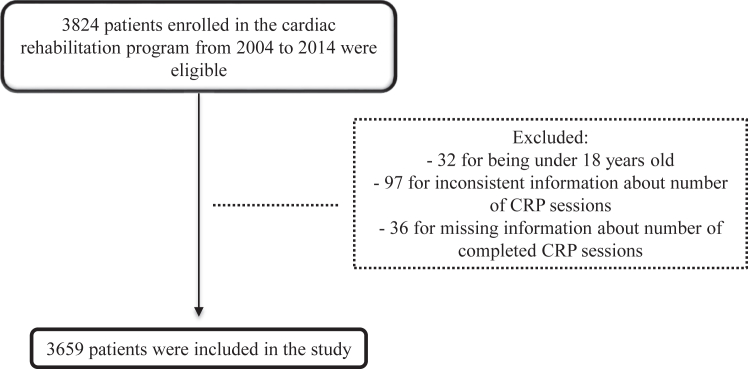
Flowchart of patient selection, illustrating the recruitment and exclusion process for the study. From an initial pool of 3824 eligible patients enrolled in the cardiac rehabilitation program (CRP) between 2004 and 2014, a total of 165 individuals were excluded. Specific reasons for exclusion included the following: age < 18 years (n = 32); inconsistent information regarding the number of CRP sessions (n = 97); and missing data on completed sessions (n = 36). Following these exclusions, 3659 patients were included in the final analysis.

We conducted a retrospective cohort study using data from the registry of participants in a clinical CRP of a high-complexity hospital in Cali, Colombia (CRP-COL). We included records of patients aged 18 years and older with a diagnosis of CVD who entered the program between January 1, 2004 and June 30, 2014. Records with inconsistent information or missing data (number of CR sessions, Hospital Anxiety and Depression Scale (HADS) scores) were excluded (Fig. 1).

### CRP-COL description

Patients admitted to the CRP were those with medical indications (eg, heart failure, or ischemic heart disease following coronary intervention) or surgical indications (eg, coronary revascularization, or valve repair or replacement) at the teaching hospital. Upon admission, sociodemographic information, health status, medical history, and HADS scores were recorded.

Subsequent CR sessions were supervised by the program's physical therapist. Each session lasted approximately 1 hour, with the duration varying according to the patients' needs. Sessions began with the measurement of baseline vital signs, followed by 15 minutes of calisthenics, 30 minutes of aerobic exercise, and finally 15 minutes of recovery. During each session, patients reported their perceived exertion using the Borg Scale, and physical activity was assessed at the end of the session. Patients also participated in educational sessions covering healthy lifestyle habits, sexual activity, cardiovascular medication use, and nutrition. At the end of the CRP, HADS measurement was administered again.^17^

### Data collection procedures

Clinical and sociodemographic data were obtained from institutional medical records and from evaluations conducted during hospitalization as part of the CRP-COL, in accordance with institutional guidelines. Additional clinical variables and cardiovascular risk factors were collected during phase II of outpatient CR (ie, the first month of intervention). These variables were defined based on clinical criteria and predefined therapeutic targets, as described in Table 1.

**Table 1:** Study variables, definitions, and data sources

Variable	Operational definition / criteria	Measurement timing	Data source
Depression and anxiety	Assessed using the HADS	Baseline and end of program	Direct questionnaire administration
Sedentary lifestyle	Failure to meet physical activity recommendations: aerobic exercise < 30 min, < 3–5 times/wk, or < 60%–80% of maximum heart rate	Baseline	Clinical interview
Dyslipidemia	Lipid levels above therapeutic targets: LDL ≥ 100 mg/dL (or ≥ 70 mg/dL in very-high-risk patients or acute coronary syndrome); triglycerides ≥ 200 mg/dL	Baseline	Medical records / initial evaluation
Hypertension	Uncontrolled blood pressure: ≥ 140/90 mm Hg; or ≥ 130/80 mm Hg in patients with diabetes mellitus, heart failure, or renal impairment	Baseline	Initial medical evaluation
Diabetes mellitus	Prior diagnosis of diabetes mellitus; inadequate control defined as fasting plasma glucose outside 80–100 mg/dL range or HbA1c ≥ 6.5%	Baseline	Medical records / laboratory findings
Smoking status	Current tobacco use (yes/no)	Baseline	Medical records / clinical interview
Nutritional status / obesity	BMI outside 20–25 kg/m^2^ and/or waist circumference > 90 cm in men and > 80 cm in women	Baseline	Initial medical evaluation
Family history of CAD	Family member with prior diagnosis of CAD	Baseline	Medical records / clinical interview

BMI, body mass index; CAD, coronary artery disease; HADS, Hospital Anxiety and Depression Scale; HbA1c, hemoglobin A1C; LDL, low-density lipoprotein.

### Definition of adherence

Adherence was defined as completion of ≥ 30 sessions (83% of prescribed sessions), a threshold supported by industry standards,^18^ published literature,^19^ and our institutional experience indicating sustained clinical benefit. This pragmatic threshold acknowledges real-world barriers while distinguishing between patients who meaningfully engaged with the program and those who discontinued prematurely.

### Confounding variables

We selected the following variables according to the published evidence and availability in the database.

#### Age

Older age is associated with higher rates of depression in cardiovascular patients due to factors such as multimorbidity and social isolation, and it may also influence CR adherence through mobility limitations or competing health priorities. Adjusting for age helps prevent overestimating the effect of depression, as younger patients with depression may be more likely to discontinue the program.^20^

#### Sex

Female patients experience higher rates of depressive and anxiety symptoms in coronary artery disease (CAD), which can hinder CR participation; however, sex differences in adherence persist independently. Including sex in the model accounts for these disparities and helps ensure that the association between depression and adherence is not confounded by sex-specific psychological burden.^21^^,^^22^

#### Body mass index (BMI)

A higher BMI, indicating obesity, is associated with poorer health-related quality of life at CR entry and may affect adherence through physical limitations. BMI is also linked to depression through pathways such as inflammation and weight-related stigma. Adjusting for BMI helps isolate the association between depression and program completion from obesity-related barriers to adherence.^23^^,^^24^

#### CAD

A history of CAD increases the risk of depression through disease burden and recurrent events and also directly influences CR referral and enrollment. If unaccounted for, CAD severity may make it appear that depression causes low adherence, when in fact both may be consequences of more severe CAD.^25^

#### Sedentary lifestyle

Sedentary behaviour is associated with greater depressive symptoms in patients with acute coronary disease, and it independently predicts lower CR engagement due to low motivation or poorer baseline fitness. Adjusting for sedentary behaviour helps clarify whether depression is driving nonadherence or whether preexisting inactivity mediates this relationship.^26^

### Statistical analysis

Quantitative variables were described using measures of central tendency and dispersion, according to the normality assumption assessed with the Shapiro–Wilk test. Categorical variables were presented as absolute frequencies and percentages. The study population was characterized according to the primary outcome and the presence of clinical levels of anxiety or depression. Anxiety and depression were assessed using the HADS, with subscale scores ranging from 0 to 21. Following established international guidelines and Spanish-language validation studies, we applied a threshold of ≥ 8 points to define clinically significant anxiety (HADS-anxiety score ≥ 8) and depression (HADS-depression score ≥ 8). This threshold has been validated in both English- and Spanish-speaking populations,^27^^,^^28^ demonstrating an optimal balance of sensitivity and specificity for identifying clinically relevant psychological distress warranting intervention.

To evaluate the association between depression or anxiety and nonadherence to CR, we first conducted a bivariate analysis. Either χ^2^ or Fisher’s exact tests were applied to categorical variables, and the Wilcoxon test was used for nonparametric continuous variables. Five multivariable logistic regression models were then constructed. First, we specified a reference model (model 0) consisting of crude odds ratios (ORs) for anxiety and depression. We subsequently developed model 1, which included depression and the confounding variables available in the database. These variables were selected based on theoretical and clinical evidence regarding their relationships with anxiety and depression and adherence. Model 2 was analogous to model 1, with anxiety substituted for depression. Model 3 included the confounding variables and both depression and anxiety simultaneously. A final model (model 4) included the confounding variables, anxiety and depression, and additional predictors selected using LASSO (for least absolute shrinkage and selection operator), a regularization technique that applies a penalty to reduce overfitting and multicollinearity, improve model performance, and identify the variables most important for predicting the outcome. For all models, odds ratios (ORs), 95% confidence intervals, and *P*-values were reported. Models were compared using information criteria (Akaike information criterion [AIC] and Bayesian information criterion [BIC]) and the likelihood ratio test (LRT). All analyses were conducted in R (version 4.5.2; R Foundation for Statistical Computing, Vienna, Austria) using the RStudio integrated development environment (version 2026.01.0).

### Ethical considerations

The study was approved by the Institutional Biomedical Research Ethics Committee (Act No. 06, March 22, 2023) and classified as minimal risk according to international standards. The requirement for informed consent was waived by the committee.

## Results

A total of 3659 patients attended the CRP-COL between January 1, 2004 and June 30^,^ 2014. The median age was 62 years (interquartile range [IQR] 53-71); most were male (66.2%); sedentary lifestyle was the most frequent risk factor (62.7%); and obesity was reported in 17.3%. The median baseline HADS anxiety score was 5 (IQR 2-8); 15.3% scored in the doubtful/mild range, and 12.6% scored in the moderate-to-severe range. The median HADS depression score was 3 (IQR 1-6); 10.9% scored in the doubtful/mild range and 5.6% scored in the moderate-to-severe range (Table 2). Overall, 51.1% of patients were nonadherent to CR.

**Table 2:** Characteristics of the study population according to adherence to a cardiac rehabilitation program at a teaching hospital in Cali, Colombia (CRP-COL)

Adherence				
Variable	Overall	Yes	No	*P*
	N = 3659	N = 1791 (48.9%)	N = 1868 (51.1%)	
Age, Y	62 (53–71)	63 (54–71)	61 (52–70)	0.032
Sex				
Male	2424 (66.2)	1218 (68.0)	1206 (64.6)	0.028
Family history of CAD	1968 (53.9)	961 (53.7)	1007 (54.1)	0.8
ND	7	1	6	
Personal history of CAD	1629 (44.6)	770 (43.0)	859 (46.1)	0.062
ND	7	2	5	
Hypercholesterolemia	1961 (53.7)	1006 (56.2)	955 (51.3)	0.003
ND	9	2	7	
Hypertriglyceridemia	1912 (52.4)	964 (53.9)	948 (50.9)	0.069
ND	11	4	7	
Diabetes mellitus	858 (23.5)	408 (22.8)	450 (24.2)	0.3
ND	7	1	6	
Hypertension	2028 (55.5)	986 (55.1)	1042 (56.0)	0.6
ND	7	1	6	
Smoking	1077 (29.5)	505 (28.2)	572 (30.8)	0.087
ND	14	2	12	
Sedentary lifestyle	2196 (62.7)	1023 (59.0)	1173 (66.2)	< 0.001
ND	154	58	96	
BMI	26.1 (24–29)	26.1 (24–29)	26.2 (24–29)	0.1
ND	7	1	6	
Categorical BMI				0.3
Underweight	76 (2.1)	38 (2.2)	38 (2.1)	
Normal	1273 (35.4)	633 (35.9)	640 (34.9)	
Overweight	1627 (45.2)	809 (45.9)	818 (44.6)	
Obesity	622 (17.3)	282 (16.0)	340 (18.5)	
ND	61	29	32	
Depression (HADS-D)	3.0 (1.0, 6.0)	3.0 (1.0, 6.0)	4.0 (1.0, 7.0)	< 0.001
Anxiety (HADS-A)	5.0 (2.0, 8.0)	4.0 (2.0, 8.0)	5.0 (2.0, 8.0)	< 0.001
Total HADS	8.0 (4.0, 14.0)	8.0 (4.0, 13.0)	9.0 (5.0, 14.0)	< 0.001
HADS-D severity				0.03
Normal (0–7)	3053 (83.4)	1524 (85.1)	1529 (81.9)	
Borderline/Mild (8–10)	400 (10.9)	175 (9.8)	225 (12.0)	
Moderate to severe (≥ 11)	206 (5.6)	92 (5.1)	114 (6.1)	
HADS-A severity				0.052
Normal (0–7)	2640 (72.2)	1325 (74.0)	1315 (70.4)	
Borderline/Mild (8–10)	558 (15.3)	253 (14.1)	305 (16.3)	
Moderate to severe (≥ 11)	461 (12.6)	213 (11.9)	248 (13.3)	
Initial SBP	116.0 (106.0–126.0)	115 (106–125)	117 (106–127)	0.1
ND	38	5	33	
Initial DBP	70 (61–77)	70 (61–76)	70 (61–78)	0.7
ND	35	3	32	
Initial heart rate	72 (63–83)	71 (62–82)	72 (63–84)	0.037
ND	34	3	31	
SBP after 20 min exercise	120 (110–130)	120 (110–130)	120 (110–132)	0.047
ND	34	3	31	
DBP after 20 min exercise	72 (63–80)	71 (63–80)	72 (63–80)	0.4
ND	35	3	32	
Heart rate after 20 min exercise	83 (72–98)	83 (72–97)	84 (72–98)	0.8
ND	35	3	32	

Values are median (quartile 1, quartile 3), n (%), or n, unless otherwise indicated.

*P*-values are from Wilcoxon rank-sum test or Pearson χ^2^ test.

BMI, body mass index; CAD, coronary artery disease; DBP, diastolic blood pressure; HADS-A, Hospital Anxiety and Depression Scale—anxiety; HADS-D, HADS-depression; ND, no data; SBP, systolic blood pressure.

Anxiety and depression scores were moderately correlated (rho = 0.54; *P* < 0.001). In the bivariate analysis, both depression and anxiety had statistically significant differences with nonadherence (*P* ≤ 0.001). Patients with and without depression or anxiety also differed significantly in cardiovascular risk factors profiles (Table 3). Significant differences by age, gender, hypertension, and sedentary lifestyle were observed when comparing groups with vs without depression or anxiety.

**Table 3:** Characteristics of the study population according to presence/absence of anxiety and depression, per the Hospital Anxiety and Depression Scale (HADS)

Variable	HADS-D		*P*	HADS-A		*P*
	Yes	No		Yes	No	
Age	64 (55–73)	61 (53–70)	< 0.001	62 (51–71)	62 (53–70)	0.366
Female sex	284 (22.8)	961 (77.2)	< 0.001	446 (35.8)	799 (64.2)	< 0.001
Male sex	346 (14.2)	2093 (85.8)	< 0.001	597 (24.5)	1842 (75.5)	< 0.001
BMI	26.272 (23.4–29–3)	26.107 (23.7–28.7)	0.3359	26.446 (23.7–29.0)	26.028 (23.6–28.7)	0.053
Personal history of CAD	332 (53.0)	1650 (54.1)	0.6154	572 (55)	1410 (53.5)	0.420
Diabetes mellitus	162 (25.9)	699 (22.9)	0.113	236 (22.7)	625 (23.7)	0.504
Hypertension	383 (61.2)	1658 (54.4)	0.0019	613 (58.9)	1428 (54.2)	0.009
Smoking	198 (31.73)	883 (29.0)	0.1757	301 (29.0)	780 (29.7)	0.705
Sedentary lifestyle	417 (69.0)	1795 (61.4)	< 0.001	682 (69.1)	1530 (60.3)	< 0.001
Hypercholesterolemia	318 (50.8)	1652 (54.2)	0.1163	559 (53.7)	1411 (53.6)	0.938
Hypertriglyceridemia	318 (50.8)	1603 (52.6)	0.4213	551 (52.9)	1370 (52.1)	0.626

Values are n (%) or median (interquartile range), unless otherwise indicated. BMI, body mass index; CAD, coronary artery disease; HADS-A, HADS-anxiety; HADS-D, HADS-depression.

To determine the predictive contribution of depression and anxiety to adherence in cardiac rehabilitation, we evaluated 5 nested models (see Table 4).

**Table 4:** Multivariate logistic regression models for association between depression or anxiety and nonadherence to a cardiac rehabilitation program at a Colombian teaching hospital (CRP-COL)

Model	Variable	OR	CI	*P*	AIC	BIC	LRT χ^2^	Variables in the model	Model specification
0	Depression	1.27	1.06–1.51	0.008	N/A	N/A	N/A	1	Crude (unadjusted)
	Anxiety	1.2	1.03–1.38	0.016	N/A	N/A	N/A	1	Crude (unadjusted)
1	Depression	1.18	1.04–1.35	0.01109	4805	4848	6.4514	6	A, S, BMI, CD, SD∗
2	Anxiety	1.1	0.99–1.22	0.0779	4808	4851	3.1081	6	A, S, BMI, CD, SD∗
3	Depression	1.16	1.01–1.33	0.02463	4806	4855	7.4076	7	A, G, BMI, CD, SD∗
	Anxiety	1.06	0.94–1.18						
4	Depression	1.14	1.00–1.31	0.006159	4803	4877	18.029	12	A, G, BMI, CD, SD, FCD, HC, DM, SM†
	Anxiety	1.06	0.95–1.19						

A, age; AIC, Akaike information criterion; BIC, Bayesian information criterion; BMI, body mass index; CD, coronary disease; CI, confidence interval; DM, diabetes mellitus; FCD, familiar coronary disease; G, hypertriglyceridemia; HC, hypercholesterolemia; LRT, likelihood ratio test; N/A, Not applicable; OR, odds ratio; S, sex; SD, sedentary; SM, smoking.

∗ Confounders.

† Confounders + predictors.

Model 0 corresponds to the crude ORs of the association between depression (OR:1.27; CI 1.06-1.51) and anxiety (OR 1.2; CI 1.03-1.38) with nonadherence to the CRP-COL.

Depression (model 1): After adjusting for confounders, depression remained a significant predictor (OR = 1.18, P = 0.011; AIC = 4805; LRT χ2 = 6.45).

#### Contribution of anxiety

Model 2, which evaluated anxiety alone, did not reach statistical significance (AIC = 4808; LRT χ^2^ = 3.11, *P* = 0.078). Notably, adding anxiety to a model that already included depression (model 3 vs model 1) did not improve model fit (LRT χ^2^ = 0.96, *P* = 0.328), suggesting that depression accounts for most of the predictive variability attributable to psychological factors.

Final model (model 4). Model 4, which incorporated additional clinical variables selected via LASSO, demonstrated the best fit (AIC = 4803). The selected variables included family history of CAD, hypercholesterolemia, diabetes, and smoking. This model supports depression as an independent predictor of nonadherence, whereas adding anxiety to a model already containing depression did not provide additional predictive value. Model 4 improved fit compared with model 3 (LRT χ^2^ = 10.62, *P* = 0.031), indicating that clinical factors contribute incremental predictive value beyond psychological variables.

## Discussion

This 10-year retrospective cohort study involving 3659 cardiovascular patients represents one of the largest investigations on predictors of CRP adherence in Latin America. Our main finding is that baseline depression is a predictor of adherence, anxiety does not add any additional value, and adding clinical factors significantly improves prediction (Central Illustration). With an adherence rate of 48.9, similar to those in other studies in Latin America,^14^^,^^17^ but lower than those in high-income countries,^15^^,^^29^ our results highlight the urgent need to systematically integrate psychological assessments into CRP in the region to guide tailored group or individual interventions.

**Central Illustration:**
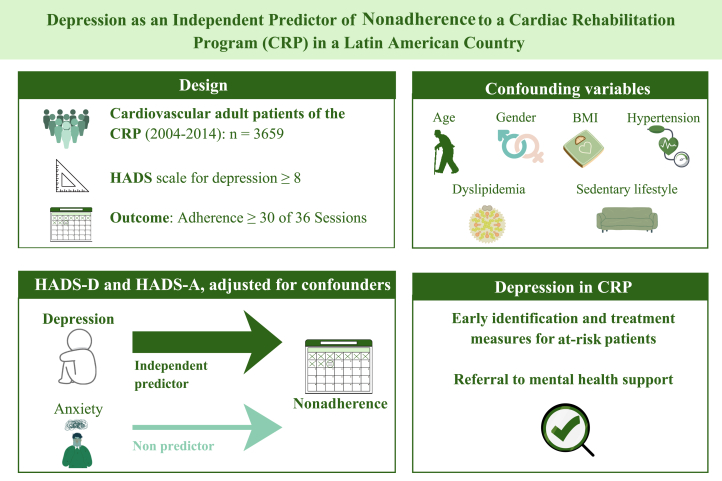
Data from 3659 patients who attended a cardiac rehabilitation program (CRP) in a Latin American country were analyzed. Baseline depression and anxiety were measured using the Hospital Anxiety and Depression Scale (HADS). Multivariable models adjusted for confounders demonstrated that depression is an independent predictor of CRP adherence (odds ratio 1.14; 95% confidence interval: 1.00-1.31), whereas anxiety does not provide additional predictive value (odds ratio 1.06; 95% confidence interval: 0.95-1.19). These findings highlight the importance of early screening and treatment of depression in CRPs. BMI, body mass index; HADS-A, HADS-anxiety: HADS-D, HADS-depression.

The interpretation of this finding is particularly relevant in the context of LMICs, where CR is available in only about 40% of countries, and both participation and adherence tend to be lower. This knowledge gap is critical, given that LMICs bear 80% of the global cardiovascular disease burden yet lack context-specific evidence on adherence predictors tailored to their socioeconomic and cultural realities.^30^, ^31^, ^32^ In Colombia specifically, limitations in health system coverage, geographic and psychosocial barriers, and differences in the management of mental health disorders may decisively influence the relationship between depression and nonadherence.

### CRP adherence and depression

Depression as a risk factor for low CRP adherence has been reported consistently across diverse contexts and populations. In our Latin American cohort, baseline depression measured by HADS was significantly associated with lower adherence. Among nonadherents, two-thirds were men, and 1 in 5 had depression. Patients with depression at CRP-COL entry were more likely to fail to complete at least 30 of the 36 scheduled sessions.

This central finding underscores the critical role of patients’ emotional state at program entry in their capacity to commit to and complete CR. Depressive symptoms can reduce motivation, energy, and optimism, making sustained participation more challenging.^29^ These results align with a 2019 meta-analysis^5^ showing that patients with depression were significantly less likely to complete a CRP, with a consequent lower likelihood of reducing cardiovascular mortality.

Our findings emphasize the need to assess not only physical but also emotional status at CR initiation. Early detection of depressive symptoms could enable medical, psychological, and social interventions aimed at improving adherence, and ultimately, CR outcomes.^29^ Even after adjusting for sedentary lifestyle, depression remained a significant predictor of nonadherence, suggesting an independent effect possibly mediated by low motivation, poor self-efficacy, hopelessness, and cognitive difficulties, such as impaired concentration or memory.^33^

These results are especially relevant in LMICs, where systematic reviews of CR effectiveness have focused mainly on clinical outcomes, with minimal evaluation of psychological factors. Given barriers such as limited access to mental health care, cultural stigma toward depression, and socioeconomic constraints, the relationship between emotional state and adherence in these settings may differ substantially from that in high-income countries.

### CRP adherence and anxiety

Although anxiety was significantly associated with nonadherence in bivariate analyses, this relationship lost significance in multivariate models after adjustment for other factors. This finding suggests that its effect may be mediated by variables such as sedentary lifestyle and hypertension, which are more prevalent in individuals with anxiety.

When depression was included in the model, anxiety’s loss of significance indicates shared underlying mechanisms, with depression exerting the stronger independent influence. Clinically, this finding suggests that directly targeting depressive symptoms may yield more impact on adherence than addressing anxiety alone. For anxiety, indirect approaches, such as promoting physical activity, and managing cardiovascular comorbidities may be more effective.^34^

### Comparing anxiety and depression

In our study, adherence rates (1 in 2 patients) fell within the lower to mid-range of international reports (39%-85%). Initially, both anxiety and depression were significantly associated with nonadherence, but only depression remained significant in multivariate models (OR 1.14; *P* = 0.006).

This finding contrasts with a retrospective case-control study in Cali, Colombia (2017-2019),^17^ which identified anxiety and not depression as the only predictor of nonadherence to the program in the multivariate analysis (adjusted OR 0.26). Differences in study design, population characteristics, inclusion/exclusion criteria, time periods (2004-2014 vs 2017-2019), and sample size (n = 3684 vs n = 993) may explain the discrepancy, highlighting the complexity of psychosocial influences on CR adherence.

A prior analysis done in the same population of our study^17^ showed that CR significantly improved affective symptoms, reinforcing the concept of a bidirectional mind–body relationship and the importance of integrating emotional care into physical rehabilitation. A 2024 review^35^ also emphasized the need to better understand adherence patterns in patients after acute myocardial infarction, to design strategies that enhance awareness and engagement.

From a theoretical standpoint, our findings support the view that psychological factors, particularly depression, are key determinants of CR adherence. Practically, they reinforce the need for systematic assessment of anxiety and depression using validated tools, such as the HADS, with early referral for patients scoring ≥ 8 on the depression subscale.

### Clinical implications

Our findings support the implementation of standardized psychological screening at CRP entry using the HADS depression subscale (cutoff ≥ 8). This approach could identify about 340 at-risk patients per 1000 admissions. Recommended interventions for such patients include early referral to mental health specialists, psychoeducation on the depression–CVD link, and identification of personal barriers.^36^ Group-based cognitive behavioral therapy, appropriate pharmacotherapy coordinated with a cardiologist, and weekly follow-up during the first 8 weeks may improve adherence and outcomes.^36^

In the Colombian context, these interventions require staff training in HADS use, clear referral pathways, protocols for severe psychiatric comorbidities, and cost-effectiveness analyses within the national health system framework.

### Limitations

First, although baseline HADS scores may not fully capture the dynamic relationship between psychological changes and adherence throughout the program, the aim of the study was to determine whether a prognostic association exists between baseline anxiety or depression and nonadherence to CRP-COL. In this sense, the authors acknowledge that both affective variables are time-varying conditions that could improve after CRP interventions. Second, given that we conducted a retrospective single-centre study, our findings may be affected by selection bias due to exclusion of patients with incomplete or inconsistent data; also, results may not be generalizable to CR settings with different cultures, resources, delivery models, or patient populations, in both Colombia and other LMICs. Third, although we adjusted for several clinical confounders, because of the clinical nature of the study, residual confounding is likely, because data on psychosocial and structural barriers (eg, socioeconomic status, transportation, work constraints, social support) were not considered. Fourth, adherence was defined solely by session attendance and did not evaluate quality of engagement or exercise performance. These limitations suggest that although depression appears to independently predict nonadherence, the magnitude of this association should be interpreted with caution, and future studies with longitudinal psychological assessments and more comprehensive psychosocial data are needed.

### Future directions

Addressing the underrepresentation of LMIC populations in CR research will require collaborative, multicentre studies that identify both universal and context-specific adherence predictors. Economic barriers, mental health access, and cultural adaptation of interventions should be explored alongside strategies such as cognitive behavioural therapy, motivational support, and digital health tools. Qualitative research can provide deeper insights into patient experiences, informing more effective, personalized, and culturally relevant programs.

## Conclusions

Depression at admission independently predicts nonadherence to CRP-COL and shows greater predictive value than anxiety. Although depression captures most of the psychological variance associated with adherence, the inclusion of clinical variables substantially improved predictive performance, confirming that adherence is a multifactorial phenomenon influenced by both psychological and clinical factors. These findings indicate that routine psychological screening may be a cost-effective strategy for Latin American CRPs, enabling early identification of high-risk patients and timely implementation of strategies to enhance participation and rehabilitation success.
